# Early weight gain as a predictor of weight restoration in avoidant/restrictive food intake disorder

**DOI:** 10.1186/s40337-024-00977-2

**Published:** 2024-02-15

**Authors:** Taylor R. Perry, Kelly Cai, David Freestone, Dori M. Steinberg, Cara Bohon, Jessie E. Menzel, Jessica H. Baker

**Affiliations:** 1 Equip Health, Inc, 2659 State Street Suite 100 #1012, Carlsbad, CA 92008 USA; 2 State University of New York at Albany, Albany, NY USA; 3 Duke University, Durham, NC USA; 4 Stanford University, Stanford, CA USA

## Abstract

**Background:**

Previous research has demonstrated that early weight gain in family-based treatment (FBT) is predictive of remission for adolescents with anorexia nervosa (AN). However, no published data has addressed if early weight gain is also predictive of reaching weight restoration (i.e., 95% EBW) in patients with avoidant/restrictive food intake disorder (ARFID). Furthermore, no studies have evaluated the performance of the statistical models used to predict weight restoration at the end of treatment. This study sought to examine whether early weight gain in ARFID is predictive of weight restoration at 20 weeks using ROC analysis. Additionally, this study assessed how accurately the model classified patients and what types of misclassifications occurred.

**Methods:**

Participants (n = 130, 57.7% cisgender female 70.0% white) received virtual outpatient FBT. Receiver operating characteristics (ROC) were used to predict successful weight restoration at end of treatment, using early weight gain as the predictor. Twenty weeks was considered as the end of treatment, to align with the definition of end of treatment in FBT clinical trials. ROC analyses demonstrated that gaining at least 6.2 pounds by week 5 of treatment was the strongest predictor of achieving 95% EBW at 20 weeks (AUC = 0.72 [0.63, 0.81]). ROC analyses misclassified 35% of patients; the most common misclassification was predicting that a patient would not achieve 95% EBW when they actually did (61.6%). A logistical regression model, which included the patients’ %EBW at admission in addition to early weight gain as a predictor, outperformed the ROC analyses (AUC = 0.90 [0.85, 0.95]) and provided additional context by showing the probability that a patient would succeed.

**Conclusion:**

Taken together, research demonstrates that early weight gain is a useful predictor of 95% EBW at 20 weeks of treatment for patients with ARFID who require weight restoration. Furthermore, results suggest that statistical models need to take into account additional information, such as %EBW at admission, along with early weight gain in order to more accurately predict which patients will reach weight restoration at week 20.

**Supplementary Information:**

The online version contains supplementary material available at 10.1186/s40337-024-00977-2.

## Abstract (plain language summary)

Results from this study indicate that when patients with avoidant/restrictive food intake disorder (ARFID) gain weight steadily at the beginning of treatment, it helps them reach 95% expected body weight (EBW) by week 20 of treatment. The more weight the patients gain each week early on, the better their chances of getting to 95% EBW by week 20. However, there are two important things to consider: how much the patients weigh when they start treatment (starting %EBW) and how much weight they gain each week. Both of these factors affect the chances of reaching 95% EBW by week 20. Thus, this study highlights the goals for gaining weight at the start of treatment need to be different for each person, depending on how much they weigh when they begin. This may help patients with ARFID reach the goal of being at 95% EBW within 20 weeks.

**Supplementary Information:**

The online version contains supplementary material available at 10.1186/s40337-024-00977-2.

## Introduction

Avoidant/restrictive food intake disorder (ARFID) was introduced under the feeding and eating disorder diagnoses in the DSM-5 [1]. ARFID is characterized by restrictive or avoidant eating due to lack of appetite, sensory sensitivity (e.g., food texture), and/or fear of aversive consequences (e.g., choking). Although being underweight is not a required diagnostic criterion, individuals with ARFID have a difficult time meeting nutritional requirements, and between 29 and 70% of patients with ARFID who presented for outpatient treatment were underweight [2–4]. Similar to anorexia nervosa (AN), low weight in ARFID has been associated with medical complications such as bradycardia [5, 6], gastrointestinal pain/dysfunction [7–11], and anemia [12–14]. Both Family-Based Treatment (FBT) for ARFID and Cognitive Behavioral Therapy for ARFID (CBT-AR) identify weight gain as a primary treatment goal for patients who require weight restoration. Since weight restoration is often a primary treatment target [3, 6], it is important to examine factors that might lead to successful weight restoration at the end of treatment for patients with ARFID.

FBT is often considered the gold standard of treatment for youth with eating disorders (EDs) and has been shown to be effective for weight gain in anorexia nervosa (AN) and for reduction in ED symptoms [15, 16]. A central aim of FBT is to empower caregivers to lead their child to recovery from the ED [17]. FBT includes three treatment phases, the first of which is weight and/or nutritional restoration [17]. Once patients reach approximately 90% of their expected body weight (EBW), they begin phase two of treatment [17]. During phase two, patients gain developmentally appropriate independence around eating. In phase three, total independence of eating begins, and the focus of treatment is on a healthy adolescent development. FBT has been adapted for ARFID [18, 19] and includes a focus on weight gain in phase one for patients who are underweight. In addition to weight restoration, FBT for ARFID emphasizes dietary expansion for patients with limited variety. A small randomized controlled trial (N = 28) demonstrated favorable weight gain for FBT in children ages 5–12 compared with treatment as usual (i.e., any medical or psychological treatment available in the community) [19]. A treatment manual on the adaptation of FBT for ARFID is also available [20]. To date, no large randomized controlled trials of FBT for ARFID have been published, although one is currently underway (Lock: R01MH121292).

A considerable amount of literature has examined factors that lead to remission at the end of treatment in FBT for patients with AN, and this work may provide useful insights into predictors of weight restoration for ARFID. In previous literature, remission for AN has been defined as being weight restored (defined as reaching 95–100% of a patient’s EBW, where EBW was the median Body Mass Index (mBMI) for that patient’s age and sex [15, 16, 21–23]) and having ED cognitions within 1 standard deviation of community norms on a symptom questionnaire. Research has continuously demonstrated that early weight gain around sessions 2–4 in FBT for AN predicts remission by end of treatment [21–25] and at 12-month follow-up [24, 25]. Furthermore, weight gain in later sessions (e.g., 5, 8, 9) has been identified as the strongest predictor of remission by week 20 [21, 23, 24]. For example, for those with AN, gaining 5.8 pounds by session 3 of FBT is the earliest predictor and 11.2 pounds by session 8 of FBT is the strongest predictor of remission at end of treatment [21]. Though studies consistently show that gaining weight early in FBT for patients with AN is a marker of remission at the end of treatment, there are variations across studies in the exact week (i.e., earliest and strongest predictors), amount of weight gain needed, and confidence in the estimates, making it challenging to translate results to practice.

While the mechanisms of food restriction in ARFID and AN are different, weight restoration is often a treatment target for both. Many patients with ARFID present as objectively underweight [26–28] and have similar levels of malnutrition as AN [27], so it is critical to examine predictors of successful weight restoration. Since research has not examined weight restoration timelines for individuals with ARFID, this study used a well established timeline for patients with AN of 20 weeks to achieve 95% EBW [21–25]. This study aimed to examine if early weight gain predicted 95% EBW at week 20 of FBT for patients with ARFID. Specifically, we determined the weeks that were the earliest and strongest predictors of reaching 95% EBW by week 20. Given that treatment for both AN and ARFID emphasize expanding dietary patterns, we hypothesized that early weight gain would be a predictor of 95% EBW at week 20 of treatment for patients with ARFID. Finally, we closely examined the statistical models used for the analysis to better understand their performance and patterns of misclassification of the analyses.

## Method

### Participants

Our initial sample included 187 patients with ARFID seeking virtual-FBT treatment from September 2020 to May 2023, who received at least 20 weeks of treatment. Seventy-six percent of these patients required weight restoration; those who did not require weight restoration were excluded from the analysis (n = 44). The analytical sample was further reduced because weight data was not available at week 0 (i.e., first week of treatment) (n = 2) or week 20 (n = 10), or because EBW was not available (n = 15).

Our final analytical sample was N = 130. Patients ranged from 5 to 29 years old (M = 14.3, SD = 4.1) and were primarily white (n = 91, 70.0%) and cisgender girls/women (n = 75, 57.7%). Average percent EBW at admission was 84.6% (SD = 7.4%). Patients, or caregivers for minors, gave informed consent for treatment data to be analyzed and disseminated for research purposes. The analysis of patient treatment outcomes was also reviewed by Western Institutional Review Board (WIRB, Puyallup, WA), an independent ethics committee. WIRB determined the evaluation of our patient outcomes does not meet the definition of human subjects research.

### Treatment overview

Patients were enrolled in a virtual ED treatment program that uses an enhanced FBT approach. The enhanced approach consists of the conventional treatment team (e.g., family therapist, registered dietitian, and medical provider), and also includes a family mentor and peer mentor. A family mentor is a caregiver who has previous experience of caring for a loved one undergoing ED treatment, and a peer mentor is an individual who has recovered from an ED. Both the family mentor and peer mentor serve as additional support for the family and patient throughout treatment. Sessions are conducted via a HIPAA compliant telehealth platform with caregivers, patients, or both. Further details on the treatment approach and effectiveness are described in detail elsewhere [29, 30].

### Measures

#### Weight

Weight was measured at home, by a family member who received in-depth training from the treatment team on weight monitoring. Patient's weight was checked two times a week with minimal clothing, after voiding, and prior to food or beverage consumption. Family members received automated prompts to enter weight into the electronic health record. EBW was determined using an individualized approach and was set by the patient's registered dietitian [31]. Dietitians calculated EBW by using each patient’s age-adjusted body mass index (BMI) and growth charts to determine where patient percentile BMI was trending before onset of the eating disorder. In addition, dietary intake, eating behaviors, physical activity patterns, medical data (vitals, blood work) and menstruation history (for those who menstruate) were used to establish EBW.

#### Weight restoration/remission

We defined weight restoration based on previous literature, such that a patient was considered weight restored if they reached 95% of their EBW by 20 weeks of treatment [16, 21, 24], a timeframe that generally aligns with how end of treatment is defined in FBT clinical trials. [16, 21–24].

### Statistical analyses

First, to describe our sample, we used a combination of t-tests and linear regressions. Weight gain over time was assessed using two multilevel linear models, one to fit weight in pounds, the other fit weight as a proportion of EBW. Both models were identical except for the outcome variable, and included the log treatment week as a term. The models also included random intercepts and slopes on log treatment week.

#### Receiver operator characteristic (ROC) analyses

We used ROC analysis in each week of treatment using weight gain as the predictor value to predict weight restoration at 20 weeks of treatment. The weight gain cutpoint chosen for each week was the one that maximized the sum of sensitivity and specificity. We report the area under the curve (AUC), its confidence intervals, and the sensitivity and specificity. We performed the analysis using weight change in pounds as well as the percent weight change from admission as the predictor and achieved similar results.

In ROC analysis, there is a binary outcome variable (in this study, weight restoration by 20 weeks), and predictor value (weight gain in a particular week) that can be used to distinguish between patients who reach weight restoration and those who do not. The cutpoint is a number that is meant to cleanly separate individuals into these two categories based on the predictor value. If a good cutpoint is found, it can be used in a clinical setting to determine whether progress early on in treatment is likely to end in weight restoration for a particular patient. For example, a weight gain cutpoint determined by the ROC analysis of 6.2 pounds in week 5 means that any patient who gains more than 6.2 pounds by week 5 is predicted to be weight restored at the end of treatment, and any patient who gains less than that is predicted not to. In this example, the cutpoint that maximizes the sum of the sensitivity (true positive rate) and specificity (true negative rate) is chosen. The ROC curve is the plot of the sensitivity against the specificity for each possible cutpoint value. The AUC characterizes how well the predictor value is able to categorize patients, regardless of the specific cutpoint. Because the ROC curve is built from a finite sample of data and possible cutpoints, the AUC we calculate is an estimate of the true AUC, and comes with uncertainty, measured by confidence intervals.

The cutpoint that maximizes the sum of sensitivity and specificity is the most common method used in the ED literature, although other methods exist. For example, if maximizing accuracy (the percentage correctly classified) was more important for the analysis, it would result in a cutpoint of 3.7 pounds in week 5. Maximizing the F1 score (a measure of accuracy based on precision and recall) would result in a cutpoint of 1 pound in week 5. The cutpoint R package contains 14 possible ways of estimating the cutpoint, and the possible cutpoints for the ROC analysis for week 5 ranged from 1 to 7.7 pounds (M = 4.9, SD = 2.1).

#### Logistic regression

Lastly, we used logistic regression in each week of treatment with a set of coefficients (the patients’ %EBW at admission, the weight gain by that week, and the interaction between the first two variables) that would predict whether a patient would be weight restored at 20 weeks of treatment. Similar to the ROC analysis, we report the AUC, its confidence intervals, and the sensitivity and specificity. These models outperformed their ROC counterparts. We report the estimated probabilities of a patient reaching 95% EBW as a function of the patient’s %EBW at admission and their weight gain in a particular week (see Table 2).

All analyses were conducted with R version 4.3.0 [32]. Fitting was done using base R (lm and glm), lme4 version 1.1-33 [33], and lmerTest packages version 3.1-3 [34]. ROC analysis was performed using pROC version 1.18.0 [35] and cutpointr version 1.1.2 [36]. Model coefficients are presented with their corresponding standard errors. The targets package version 0.14.2 [37] was used for project management.

## Results

### Patient characteristics

**Fig. 1:**
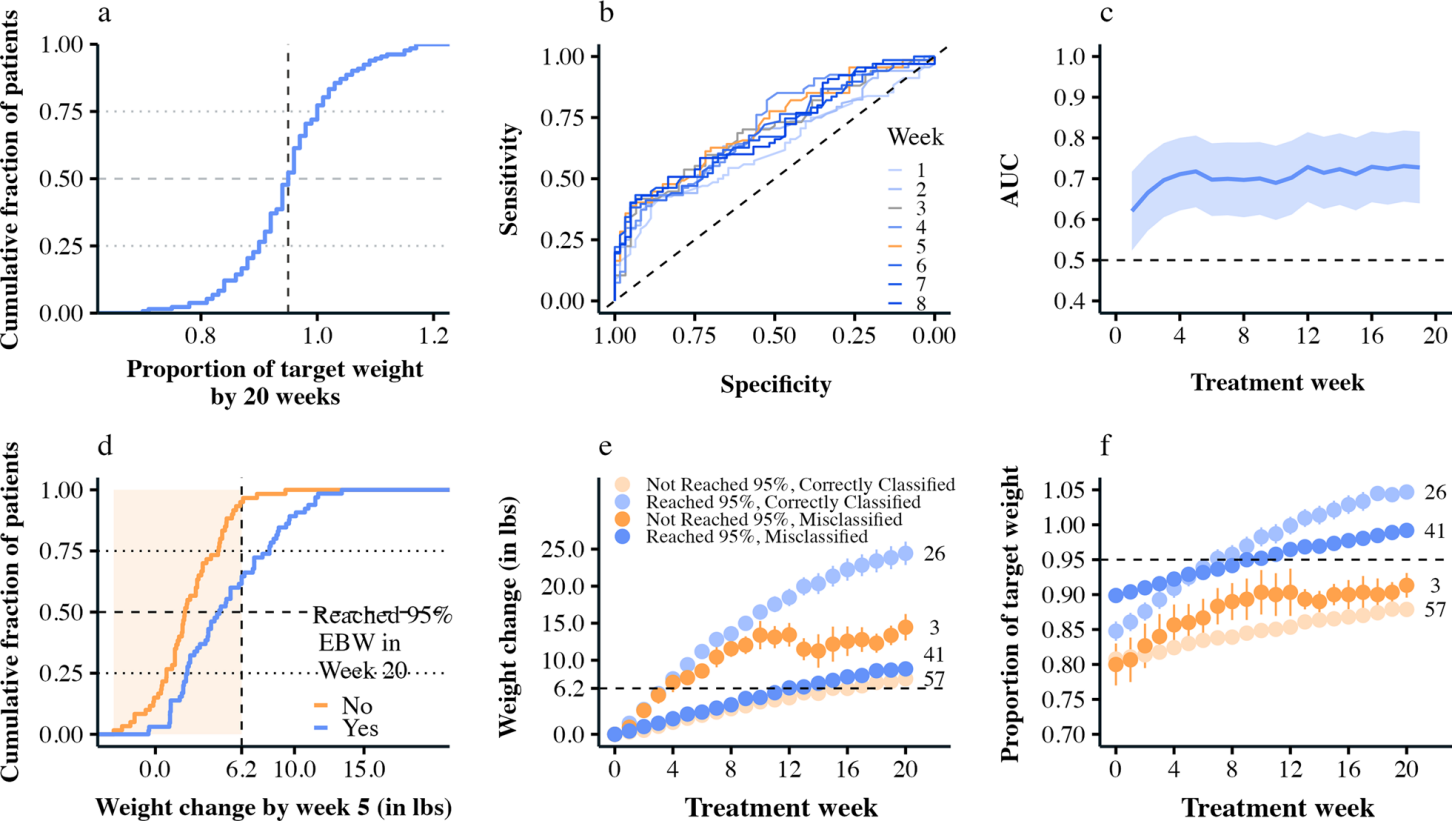
ROC classification and misclassification. **a** Every patient’s weight in week 20, expressed as a proportion of EBW (x-axis), against the cumulative fraction of patients (y-axis). The horizontal dashed lines at 0.25, 0.50, and 0.75 give the lower quartile, median, and upper quartile of the distribution. The average and median %EBW by 20 weeks is roughly 95%, with about half the patients being higher, and half lower. **b** The ROC curves using weight change in the first 8 weeks of treatment from lighter (week 1) to darker (week 8) shades of blue. The curve with the highest AUC is shown in orange, and corresponds to week 5 of treatment. **c** The AUC for the ROC models using weight change in weeks 1 through 19 of treatment. Aside from the initial increase in predictive performance, the ROC model’s AUC hovers around 0.70 (dark blue line) with wide confidence intervals (shaded blue ribbons). **d** The empirical cumulative distribution of weight change in week 5, grouped by patients who reached 95% of their target (blue) and those who did not (orange). The vertical dashed line represents the weight gain cutpoint determined by the ROC analysis for week 5, such that anyone over 6.2 was predicted to reach 95% EBW, and anyone under was predicted not to. **e** The average (+−se) weight change from admission in each week for patients grouped by whether the ROC analysis correctly or incorrectly classified them using the cutpoint in week 5. The dark blue line are patients who reached the target, but were predicted not to, and the dark orange line are patients who did not reach the target, but were predicted to. The lighter lines are cases where the ROC algorithm correctly predicted the outcome. The number of patients in each category is printed. **(f)** plots the average (+−) weight in each treatment week as a proportion of EBW. The horizontal dashed line gives 95% EBW

Patients with AFRID started treatment at 92 pounds (SD = 25.6) on average and needed to gain 16.9 pounds (SD = 9.6) on average to be weight restored. By 20 weeks of treatment, almost exactly half of the patients reached 95% of their EBW (Fig. 1a). Their weight (expressed as a proportion of their target) by week 20 is normally distributed; most patients clustered around 95% of their EBW after 20 weeks of treatment.

On average, patients gained weight logarithmically over time (b = 4.1 ± 0.29, *p* < 0.001). Patients gained more weight in the beginning of treatment, with weight gain slowing as treatment progressed. Growth trajectories varied by patient (some faster, some slower), and were better classified as a continuous curve (random effects SD = 3.4) rather than into classes like “slow” and “fast” progress. When weight was expressed as a proportion of the patient’s EBW, the rate of change was more linear (b = 0.04 ± 0.003, *p* < 0.001).

### ROC analyses

ROC analyses showed that weight change in sessions 1–8 was significantly associated with reaching 95% EBW in week 20 (Fig. 1b, c). AUC values ranged from 0.62 to 0.72 for weeks 1–8 (see Table 1). The earliest predictor was increasing weight by 1.2 pounds from the start of treatment (i.e., week 0) to week 1 of treatment (AUC = 0.62 [0.52, 0.72], sensitivity = 0.43, specificity = 0.85), and the strongest predictor was increasing weight by 6.2 pounds from the start of treatment to week 5 (AUC = 0.72 [0.63, 0.81], sensitivity = 0.39, specificity = 0.95).

**Table 1:** ROC analysis, success = reached 95% EBW in week 20

Treatment week	n	AUC (95% CI)	Weight gain cutpoint (pounds)	Sensitivity	Specificity
1	130	0.62 (0.52–0.72)	1.20	0.43	0.85
2	130	0.67 (0.57–0.76)	2.25	0.40	0.90
3	127	0.70 (0.61–0.79)	3.60	0.42	0.93
4	128	0.71 (0.62–0.80)	1.60	0.82	0.52
5	127	0.72 (0.63–0.81)	6.20	0.39	0.95
6	130	0.70 (0.61–0.79)	7.50	0.38	0.95
7	129	0.70 (0.61–0.79)	8.05	0.43	0.94
8	125	0.70 (0.61–0.79)	9.70	0.40	0.95

#### Misclassification

Some patients were misclassified by the ROC analysis, such that the predicted outcome did not match the actual outcome for that patient. The weight gain cutpoint determined by the ROC analysis in week 5 (the strongest predictor of achieving 95% EBW; Fig. 1d–f) misclassified 34.6% of patients. The four possible outcomes were that (1) the model correctly predicted that a patient would achieve 95% EBW by 20 weeks (n = 26), (2) the model correctly predicted that a patient would not achieve 95% EBW (n = 57), (3) the model incorrectly predicted that a patient would achieve 95% EBW (n = 3), and (4) the model incorrectly predicted that a patient would not achieve 95% EBW (n = 41). The model made the fewest errors when predicting that a patient would succeed (3/29 = 10.3% false discovery rate), and made the most errors when classifying the patients who actually did succeed (41/67 = 61.1% false negative rate). Figure 1f helps explain why. Patients who did not achieve 95% EBW (light and dark orange) started at a lower %EBW at admission on average. The ROC model mispredicted the cases where the patients started at a lower %EBW, but also gained weight rapidly (dark orange). Patients who did achieve 95% EBW (light and dark blue) started at a higher %EBW at admission. In this case, slower weight gain could still lead to 95% EBW by 20 weeks (dark blue). However, the cutpoint determined by the ROC analysis was high (6.2 pounds at week 5), so these patients were misclassified. Individual patient weight trajectories are given in additional file 1: S1.

### Models that included %EBW at admission

The pattern of misclassified cases shown in Fig. 1e, f suggests that weight gain alone may not convey enough information to accurately predict whether a patient will reach 95% EBW. In addition to weight gain, it may be helpful to consider the patient's %EBW at admission. With this in mind, we fit a series of logistic regression models, one for each week of treatment, that included the patient’s %EBW at admission, the weight gain in that week, and their interaction to predict the probability of a patient reaching 95% EBW in week 20. This method is analogous to ROC analysis, but can include more than one variable as a predictor, in this case, early weight gain and how far the patient was from their EBW at the start of treatment. This approach outperformed the ROC analysis (week 5 AUC = 0.90 [0.85, 0.95], sensitivity = 0.85, specificity = 0.8).

Table 2 shows the estimated probability (with standard errors) of reaching 95% EBW in week 20 based on admission %EBW and weight gained by week 5 in treatment. Results for additional weeks are shown in additional file 1: S2. Across the columns are various weight gain cutpoints (in pounds), along the rows are various starting proportions of EBW, and each cell gives the estimated probability of reaching 95% EBW in week 20. For example, a patient who starts treatment at 75% EBW and gains 6 pounds by 5 weeks has a 20 ± 10% chance of reaching 95% EBW in week 20, but a patient who starts treatment at 85% EBW and gains 6 pounds by week 5 is 74 ± 7% likely.

**Table 2:** Logistic regression model—probability of reaching 95% EBW in week 20 (P (SE))

% EBW at admission	Weight gained by week 5				
	3 pounds	4 pounds	5 pounds	6 pounds	7 pounds
75%	0.019 (0.02)	0.044 (0.03)	0.097 (0.06)	0.20 (0.1)	0.37 (0.15)
80%	0.10 (0.05)	0.18 (0.07)	0.30 (0.09)	0.46 (0.1)	0.62 (0.1)
85%	0.39 (0.08)	0.51 (0.08)	0.63 (0.08)	0.74 (0.07)	0.82 (0.07)
90%	0.79 (0.06)	0.83 (0.06)	0.87 (0.05)	0.90 (0.05)	0.93 (0.05)

## Discussion

This study sought to evaluate early weight gain as a predictor of achieving 95% EBW at week 20 in a large naturalistic virtual outpatient setting of patients with ARFID. When we performed ROC analyses using statistical methods similar to previous research, which only used early weight gain and did not account for %EBW at admission, results were consistent with previous research in AN [21–25]. Early weight gain in ARFID predicted achieving 95% EBW at week 20. Specifically, gaining 1.2 pounds in week 1 of treatment was the earliest predictor, and gaining 6.2 pounds by week 5 was the strongest predictor of achieving 95% of EBW. However, this analysis misclassified patients in two ways: predicting a patient would *not* reach 95% EBW by week 20 when the patient *did,* and predicting a patient *would* reach 95% EBW by week 20 when the patient *did not*. Logistic regression models that accounted for admission %EBW outperformed the ROC analyses and predicted more patients correctly. Taken together, results support that early weight gain is a useful predictor of 95% EBW at 20 weeks of treatment for patients with ARFID who require weight restoration. Results are in line with research that shows that greater behavioral changes early in treatment predict better treatment outcomes [38].

### Performance of ROC analyses

Predictors of treatment outcomes are only as useful as how accurately they can predict outcomes and classify patients. When using ROC analysis, approximately 35% of patients were misclassified. A majority of the errors were patients who reached 95% EBW when they were predicted not to; these patients had fewer pounds to gain to reach EBW, and fell below the cutpoint determined by the ROC analysis. ROC analyses most accurately predicted that patients would not achieve 95% EBW if they had a higher amount of weight to gain to reach EBW and were gaining at a slower rate. Given the prevalence of misclassifications, the strongest and earliest predictors from the ROC analysis should not be taken as rules, but rather serve as a guide.

Misclassification may occur due to the way ROC analysis classifies patients categorically (either above 95% EBW or below 95% EBW in week 20). The %EBW is a continuous variable, not categorical. In fact, the %EBW at 20 weeks for patients in our sample was normally distributed around 95%. The categorical classification does not provide any additional context for the predictions; this is especially relevant for patients who are achieving early weight gain cutpoints, but are just short of reaching 95% EBW (e.g., patients at 94.9% EBW in week 20). Furthermore, weight progress fluctuates. Depending on the predictive week chosen, a patient could exceed the cutpoint determined by the ROC analysis in one week of treatment, but not in the next.

### Early weight gain models including admission %EBW

In response to the pattern of misclassifications by the ROC analyses, we fit additional logistic regression models that used the patient’s %EBW at admission in addition to weight gain as a predictor. These models outperformed the ROC analysis and provided additional context by showing the probability that a patient would end up reaching 95% EBW at 20 weeks of treatment, as opposed to just predicting that a patient would or would not reach it. These results showed that the probability of reaching 95% EBW in week 20 was different for patients who had different %EBW at admission, even if they gained the same amount of weight by a certain week.

These results indicate that %EBW at admission is an important variable to consider when predicting weight restoration, and that the same cutpoint should not be used for all patients. The logistic regression model provides a more individualized way to predict patient success, and can aid providers in more accurately assessing probable weight target outcomes. Logistic regression also provides a simple framework that would allow for the inclusion of additional variables in future studies, which may further improve predictions. Future research should examine the clinical implications of these findings. Do patients who start treatment with a lower %EBW need to gain more weight early in treatment to increase their likelihood of achieving 95% EBW at 20 weeks, or do they need a longer course of treatment, beyond 20 weeks, to achieve 95% EBW?

### Clinical implications

Results from this study underscore the significance of early weight gain in the treatment of patients with ARFID requiring weight restoration. However, it is crucial to approach the establishment of early weight gain targets with caution when informed by ROC analyses, given that these benchmarks often misclassify who will reach 95% EBW by week 20. Instead, the determination of early weight gain targets should take into consideration the patient's admission EBW. For example, a patient starting treatment at 75% EBW and gaining 7 pounds by week 5 demonstrates a 35% chance of attaining 95% EBW by week 20, while a patient starting treatment at 90% EBW and achieving the same 7-pound weight gain by week 5 has a 93% likelihood of reaching the 95% EBW in week 20. Thus, clinicians should adopt an individualized approach to establish weekly weight gain goals and tailor them to the patient's %EBW at admission.

### Strengths and limitations

This study is not without limitations. Patients in treatment are not always able to provide complete data. Some patients provide weight measurements more consistently than others, and this may lead to varying types of sampling biases. An important part of our analysis was to determine the ways in which a simple ROC analysis misclassified individual patients, and handling missing data in this case is complex. Imputing missing values with a statistical procedure (i.e. MICE) may help reduce bias in the ROC cutpoints in some cases, but may not necessarily significantly help in our case. The cutpoint estimates in our results and the variability in those estimates were consistent with previous literature. Additionally, the purpose of our misclassification analysis was to evaluate the estimates from the ROC analysis against a patient’s actual outcomes. For these reasons, we chose to analyze the data that were available to us, rather than imputing or inferring missing values. However we recognize the limitations imposed by the missing data and our decisions around them.

We collapsed ARFID diagnoses across presentations into a homogeneous sample, but research supports that ARFID presentations are distinct and heterogeneous [39]. Future research should evaluate if differences in early weight exist across presentations. In particular, patients with the fear of aversive consequences presentation are most likely to present to treatment at lower weights and with more recent, acute weight loss compared to other presentations [39, 40]. Our study used an individualized target weight approach, making it difficult to compare to randomized control trials in AN that used the 50th percentile BMI as a target weight [21–25]. We only examined one aspect of recovery (i.e. weight restoration); our data does not speak to whether early weight gain is associated with changes in cognitive symptoms of ARFID. In addition, we used week 20 as the evaluation point for outcomes, which corresponds to when the majority of patients complete FBT as described in clinical trials [21–25], but may not always correspond to the end of treatment in naturalistic settings as patients may stay longer to meet treatment goals. Finally, the sample was mostly young white females receiving FBT, so results may not generalize to non-white patients, adults, or other treatment modalities.

Despite these limitations, this study has several strengths. We used statistical methods to evaluate the ability of ROC analysis to accurately predict treatment outcomes. We provided results from alternate models that had better predictive accuracy. Finally, we had a large sample size that was comparable in size to similar evaluations of early weight gain in FBT for AN.

## Conclusions

Early weight gain can be a useful metric to include in predictions of achieving 95% EBW by week 20 for patients with ARFID. However, including %EBW at admission in addition to weight gain in predictive models led to higher predictive accuracy. Early weight gain targets should take into account admission %EBW in outpatient samples of patients with ARFID, although more research is needed to determine the clinical implications of these findings. Results also need to be extended across other treatment modalities (e.g. Cognitive Behavioral Therapy for ARFID (CBT-AR)) and across ARFID presentations.

### Supplementary Information

**Additional file 1. Supplementary Material:** Individual patient weight trajectories

## Abbreviations

FBT: Family-based treatment
AN: Anorexia nervosa
ARFID: Avoidant/restrictive food intake disorder
EBW: Expected Body Weight
