# Supplementary material for: Early weight gain as a predictor of weight restoration in avoidant/restrictive food intake disorder
Source: J Eat Disord. 2024 Feb 15;12:27. doi: 10.1186/s40337-024-00977-2 (PMC10870495; doi:10.1186/s40337-024-00977-2)
Supplement: Supplementary file 1 — Additional file 1. Supplementary Material: Individual patient weight trajectories [file 40337_2024_977_MOESM1_ESM.docx]

**Additional file 1**

***S1. Misclassification for ROC analysis***

Figure S1.1 shows the same data as Figure 1f in the main text, but with individual patient trajectories broken out rather than shown as an average. This gives a better sense of the variation in trajectories and %EBW at admission for patients in each group, as well as how close some patients are to reaching 95% EBW at week 20.

| 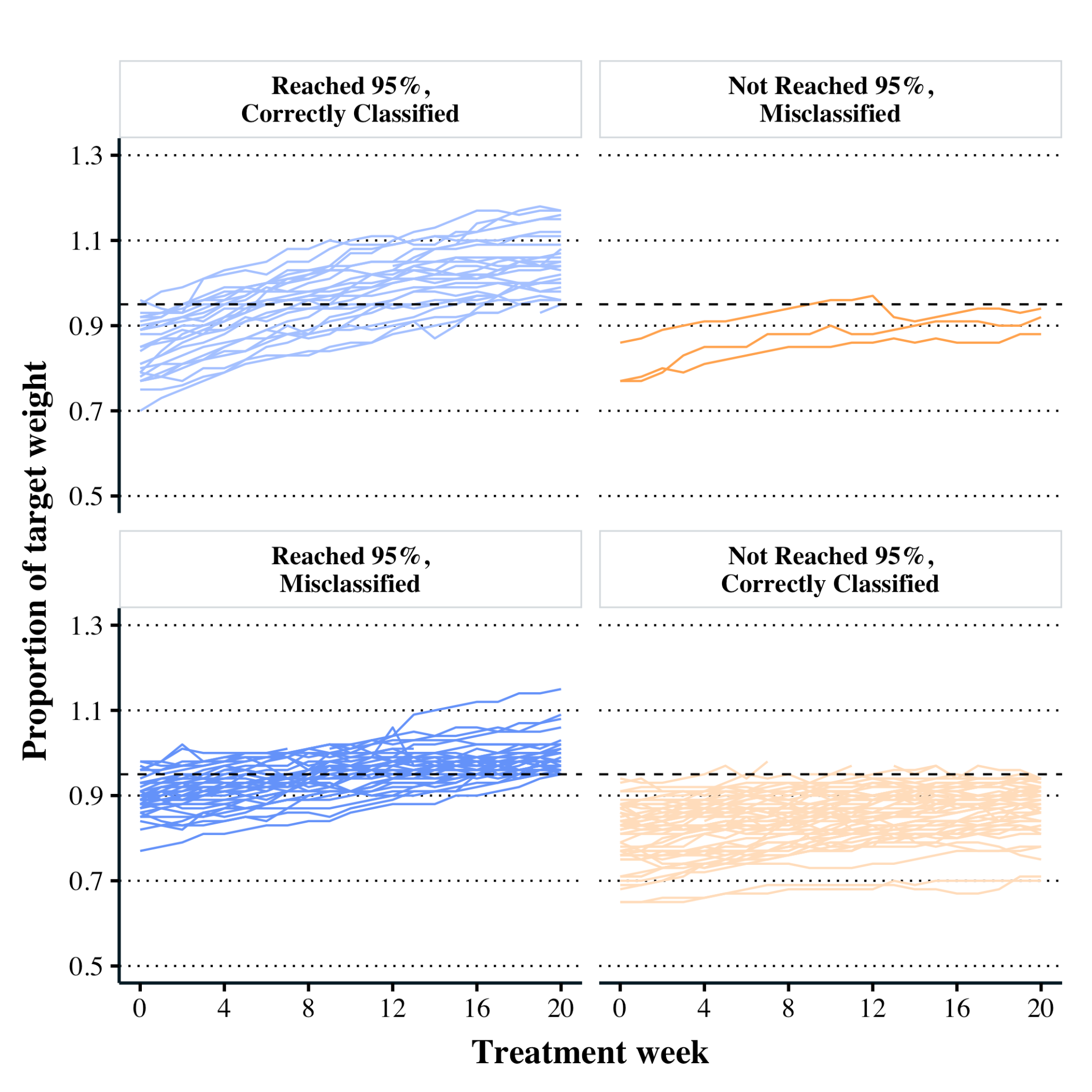 |
| --- |
| ***Figure S1.1. ROC misclassification individual patient trajectories.*** Individual trajectories of patients based on whether the ROC analysis correctly or incorrectly classified them at week 5. The horizontal dashed line gives 95% of EBW. This is the same plot as Figure 1f in the main text, but with the individual patients plotted separately as opposed to plotting the average. |

***S2. Additional results for models that include the starting % EBW***

Table S2.1 gives the estimated probability that a patient will achieve 95% EBW based on both their weight gain (columns) and their starting %EBW (columns). As described in the main text, a series of logistic regression models were run for each week of treatment (weeks 1 - 19), which took the following form

$$log\frac{p_{i}}{1-p_{i}}=\beta_{0}+\beta_{1}\%EBW_{i,0}+\beta_{2}Gain_{i,n}+\beta_{3}(\%EBW_{i,0}\times Gain_{i,n})$$

Where $log\frac{p_{i}}{1-p_{i}}$ is the log odds that patient i will achieve 95% EBW by week 20, %EBW_i,0_ is the patient’s starting %EBW, and Gain_i,n_ is the weight gained by the patient i by week n. The estimated probabilities are formed by transforming the log odds into a probability

$$P = \frac{e^{\beta_{0}+\beta_{1}\%EBW_{i,0}+\beta_{2}Gain_{i,n}+\beta_{3}(\%EBW_{i,0}\times Gain_{i,n})}}{1 + e^{\beta_{0}+\beta_{1}\%EBW_{i,0}+\beta_{2}Gain_{i,n}+\beta_{3}(\%EBW_{i,0}\times Gain_{i,n})}}$$

The coefficients we found for week 5 were 𝛽_0_=-37.5 (8.7), 𝛽_1_=42.1 (9.9), 𝛽_2_=3.2 (1.4), and 𝛽_3_=-3.2 (1.7). The worst of these series of models (week 1 AUC = 0.83 [0.76, 0.90]) still outperformed the best ROC model (week 5, AUC = 0.72 [0.63, 0.81]).

| **Table S2.1: Logistic Regression Model – Probability of Reaching 95% EBW in week 20 (P (SE))** | | | | | | |
| --- | --- | --- | --- | --- | --- | --- |
| **Weight Gained by Week** | **% EBW at Admission** | **3 pounds** | **4 pounds** | **5 pounds** | **6 pounds** | **7 pounds** |
| **3** | 75% | 0.152 (0.07) | 0.314 (0.11) | 0.539 (0.16) | 0.749 (0.16) | 0.884 (0.11) |
|  | 80% | 0.353 (0.08) | 0.535 (0.09) | 0.708 (0.09) | 0.836 (0.08) | 0.915 (0.06) |
|  | 85% | 0.624 (0.07) | 0.743 (0.07) | 0.834 (0.06) | 0.897 (0.05) | 0.938 (0.04) |
|  | 90% | 0.835 (0.05) | 0.879 (0.05) | 0.912 (0.05) | 0.937 (0.05) | 0.955 (0.04) |
| **4** | 75% | 0.064 (0.04) | 0.125 (0.06) | 0.229 (0.09) | 0.383 (0.13) | 0.564 (0.16) |
|  | 80% | 0.206 (0.07) | 0.32 (0.08) | 0.46 (0.09) | 0.607 (0.09) | 0.737 (0.09) |
|  | 85% | 0.496 (0.07) | 0.608 (0.07) | 0.71 (0.07) | 0.794 (0.07) | 0.859 (0.06) |
|  | 90% | 0.789 (0.06) | 0.837 (0.06) | 0.875 (0.06) | 0.906 (0.05) | 0.93 (0.05) |
| **5** | 75% | 0.025 (0.02) | 0.054 (0.03) | 0.112 (0.06) | 0.218 (0.1) | 0.379 (0.15) |
|  | 80% | 0.117 (0.05) | 0.199 (0.07) | 0.318 (0.08) | 0.466 (0.1) | 0.62 (0.1) |
|  | 85% | 0.402 (0.07) | 0.518 (0.07) | 0.631 (0.07) | 0.732 (0.07) | 0.814 (0.07) |
|  | 90% | 0.773 (0.06) | 0.823 (0.06) | 0.863 (0.05) | 0.896 (0.05) | 0.921 (0.05) |
| **6** | 75% | 0.015 (0.01) | 0.028 (0.02) | 0.054 (0.03) | 0.102 (0.05) | 0.183 (0.08) |
|  | 80% | 0.08 (0.04) | 0.131 (0.06) | 0.208 (0.07) | 0.314 (0.08) | 0.444 (0.09) |
|  | 85% | 0.339 (0.07) | 0.44 (0.07) | 0.547 (0.08) | 0.65 (0.08) | 0.74 (0.08) |
|  | 90% | 0.751 (0.06) | 0.803 (0.06) | 0.847 (0.06) | 0.882 (0.05) | 0.91 (0.05) |
